# Simulation and Experimental Study of Non-Resonant Vibration-Assisted Lapping of SiCp/Al

**DOI:** 10.3390/mi15010113

**Published:** 2024-01-09

**Authors:** Huibo Zhao, Yan Gu, Yuan Xi, Xingbao Fu, Yinghuan Gao, Jiali Wang, Lue Xie, Guangyu Liang

**Affiliations:** 1 Jilin Provincial Key Laboratory of Micro-Nano and Ultra-Precision Manufacturing, School of Mechatronic Engineering, Changchun University of Technology, Yan’an Ave 2055, Changchun 130012, China; zhaoxx7618@163.com (H.Z.); 13944475509@163.com (Y.X.); 15568607008@163.com (X.F.); gyh101x@163.com (Y.G.); 15968441655@163.com (J.W.); xl1280530@163.com (L.X.); liangguangyu2023@163.com (G.L.); 2 Jilin Provincial Key Laboratory of International Science and Technology Cooperation for High Performance Manufacturing and Testing, School of Mechatronic Engineering, Changchun University of Technology, Yan’an Ave 2055, Changchun 130012, China

**Keywords:** non-resonant vibration-assisted lapping, SiCp/Al, vibration-assisted lapping device, surface integrity

## Abstract

SiCp/Al is a difficult-to-machine material that makes it easy to produce surface defects during machining, and researchers focus on reducing the surface defects. Vibration-assisted machining technology is considered an effective method to reduce surface defects by changing the trajectory and contact mode of the abrasive. Aiming at the problem of SiCp/Al processing technology, a vibration-assisted lapping device (VLD) is designed, and elliptical motion is synthesized by a set of parallel symmetrical displacement output mechanisms. The working parameters of the device were tested by simulation and experiment, and the lapping performance was verified. Then, the effects of removal characteristics and process parameters on surface roughness and lapping force were analyzed by simulation and experiment. Simulation and experimental results show that frequency and amplitude that are too low or too high are not conducive to the advantages of NVL. The best surface quality was 54 nm, obtained at A = 8 μm and f = 850 Hz.

## 1. Introduction

SiCp/Al has broad application prospects and excellent material properties, and its special strengthening mechanism makes it easy to form surface defects during processing [1,2]. The high hardness of SiC particles in SiCp/Al will increase tool wear and reduce processing efficiency and processing economy [3,4]. At the same time, particle breakage, particle fracture, and interfacial debonding will occur in the process of SiC particles; these phenomena constitute the main form of its surface defects.

The abrasive in the conventional lapping (CL) method is in continuous contact with SiCp/Al, and the strength difference between the aluminum matrix and SiC particles leads to surface damage of SiC particles during processing, so there are large heating and pits in the processing process [5,6]. Many studies have pointed out that ultrasonic vibration-assisted machining (UVM) technology and non-resonant vibration-assisted machining (NVM) technology can reduce force and surface damage by changing the motion path of the abrasive or tool [7,8,9]. Vibration-assisted machining technology based on ultrasonic vibration devices improves surface quality and surface integrity by making abrasive or tools hit the workpiece surface at high frequency [10]. This technique has received much attention [11,12,13]. Gu et al. predicted the surface roughness of SiCp/Al by ultrasonic vibration-assisted lapping (UVL). The experimental results verified the effectiveness of UVL in improving surface roughness [14]. Zheng et al. compared the material removal, friction coefficient, and scratch force behavior between the CL method and the UVL method through scratch experiments. The experimental results show that the UVL reduces the scratch force and friction coefficient [15]. Li et al. carried out an ultrasonic vibration-assisted scratch experiment and analyzed the primary forms of sub-surface damage. The experimental results showed that the UVM method had an inhibiting effect on sub-surface damage. According to the above results, NVM can help improve surface quality and reduce surface defects [16]. Wu et al. designed a rotary ultrasonic lapping spindle and carried out SiCp/Al machining experiments. Observing and analyzing the surface morphology of SiCp/Al verified that the axial UVM method can also reduce the lapping force and surface roughness [17].

Gu et al. established a surface roughness prediction model of UVL with a maximum error of 3.64% and obtained the optimal process parameters through the roughness prediction model [13]. Therefore, appropriate process parameters are essential in optimizing surface quality [18]. Although UVL has a series of advantages compared to CL, it also has some unavoidable problems. The ultrasonic vibration device usually works near the resonance point, and its high-frequency vibration will produce much heat, affecting the processing effect of SiCp/Al composite materials [19,20]. In addition, resonant ultrasonic vibration suffers from many limitations in vibration frequency and amplitude adjustment, which makes it difficult to achieve a wide range of free adjustments, and the selection of processing parameters will be limited [21,22].

Researchers have tried to apply the non-resonant vibration device to vibration-assisted cutting (VC) [23,24], vibration-assisted milling (VM) [25], and vibration-assisted polishing (VP) [26,27] and achieved good results. Zhou et al. designed an elliptical VC device and studied the removal mechanism, cutting force, surface roughness, and microstructure in elliptical VC. The orthogonal cutting experiments showed that the periodically changing cutting trajectory reduced the average cutting force and improved the surface quality [24]. Zheng et al. showed that applying vibration on a micro-milling cutter can help improve tool life, and this method can improve surface roughness. The above research results demonstrate the positive effect of non-resonant vibration-assisted machining (NVM) on improving surface quality and reducing surface defects [25]. Gu et al. proposed a non-resonant VP method to investigate the effect of plane vibration on machining processes. Research showed that introducing plane vibration changes the abrasive trajectory and force, thereby improving the abrasive removal capability [26,27]. Wang et al. designed a three-dimensional VP device for structured surface polishing. Combined with the workpiece surface and two-dimensional vibration parameters, the three-dimensional space vibration trajectory and polishing trajectory model were established. The influence of phase and frequency on the polishing trajectory was analyzed by simulation. The experimental results proved the feasibility and effectiveness of three-dimensional VP technology for structured surface polishing [28].

In the NVM method, by adjusting the amplitude and frequency, the tool moves in a specific trajectory, making the tool or the workpiece periodically separated [29], and the contact mode of abrasive is changed from continuous contact to periodic contact so that the processing force is reduced, and the chips can be discharged quickly. Although the NVM method has been introduced into many fields, the processing mechanism and technology of non-resonant vibration-assisted lapping (NVL) SiCp/Al are not fully studied. The key to obtaining better surface quality and surface integrity in the processing of SiCp/Al is to avoid large area fracture and pulling off of SiC particles, so amplitude and frequency processing parameters are essential.

This paper mainly analyzes the removal characteristics and processing technology of NVL SiCp/Al. Firstly, a two-dimensional VLD was designed, and its performance was simulated and tested. Then, a single abrasive NVL SiCp/Al finite element model was established. Based on the relationship between the tool path and the relative position of silicon carbide, the removal characteristics of SiCp/Al during NVL were discussed. Finally, the effects of spindle speed *n*, feed speed *v_f_*, lapping depth *a_p_*, amplitude *A*, and frequency *f* on lapping force and surface quality were studied by simulation and experiment methods.

## 2. Principle of Processing

**Figure 1:**
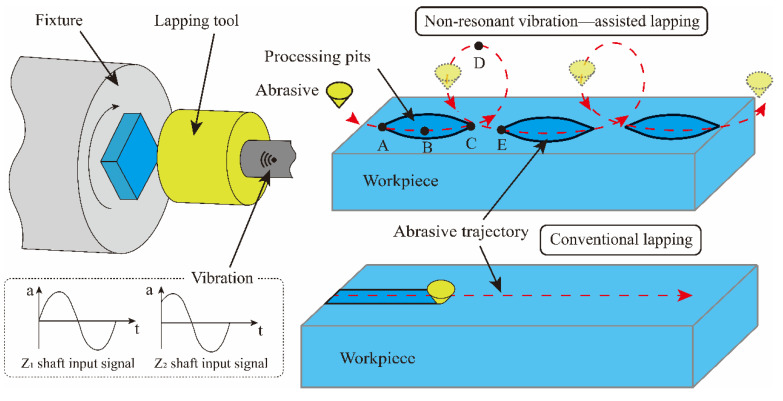
Schematic diagram of non-resonant vibration-assisted lapping.

The characteristic of NVL SiCp/Al is that the lapping tool moves in the space in the form of elliptical vibration, and the formation of the lapping tool trajectory depends on the output signal of the VLD. Figure 1 shows the motion path of abrasive lapping in the NVL and CL methods. In the traditional lapping method, the abrasive keeps continuous contact with the machined workpiece under pressure, and the higher contact pressure can easily cause surface damage to the workpiece, which is not conducive to improving the Surface quality [30]. In the vibration-assisted lapping method, the motion path of the abrasive changes due to the application of vibration. Abrasive passes through points A, B, C, D, and E. The abrasive is pressed into the material at point A, reaches the lowest point of the trajectory at point B, and separates from the workpiece at point C and point D is the highest point of the processing trajectory, and one cycle of processing is completed when point E is reached.

## 3. Design and Analysis of VLD

**Figure 2:**
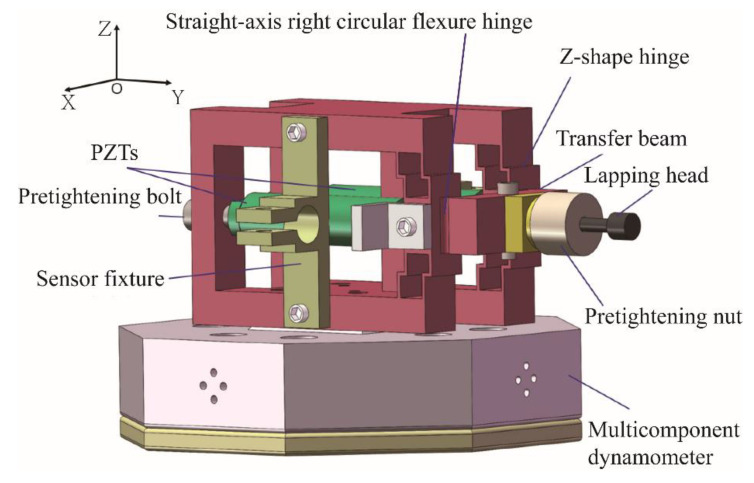
Assembly diagram of vibration-assisted lapping device.

The structural model of VLD is shown in Figure 2. The VLD comprises Z-hinge groups, a straight-axis right circular flexure hinge, a piezoelectric actuator (PZT), a tightening bolt, and a lapping tool. The device is symmetric with respect to the XOZ and the XOY plane, and the two PZTs are placed in parallel. Through the transmission of the Z-shape flexible hinge group and straight-axis right circular flexure hinge, the output displacement will be coupled at the transfer beam. Finally, the movement trajectory will be output at the end of the lapping tool. A fixture that holds the displacement sensor is fixed on the displacement output mechanism, and the VLD is set on the multicomponent dynamometer. Such a structure not only ensures the stability of the device but also ensures that the VLD has no output displacement in the longitudinal direction.

### 3.1. Vibration Trajectory Analysis

**Figure 3:**
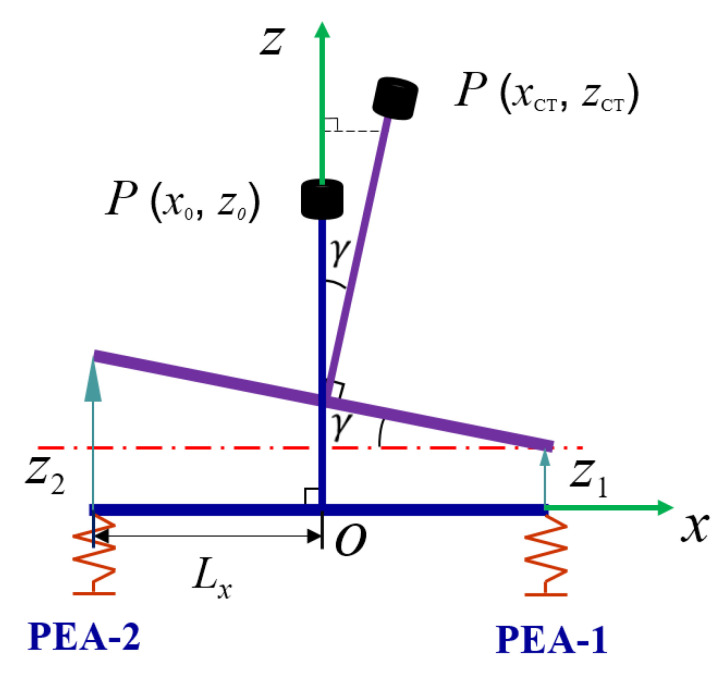
The schematic diagram of device movement.

By feeding electrical signals with phase differences to the two piezoelectric actuator sets, the piezoelectric actuator’s output displacement can synthesize an elliptical motion. In the kinematic analysis, the lapping tool and transfer beam are assumed to be rigid T-bars, which do not deform during processing. As depicted in Figure 3, define the coordinate system O−xz for reference. Within this coordinate system, $P\left(x_{0},z_{0}\right)$ signifies the equivalent coordinate of the lapping head in the absence of input displacement. Furthermore, $P\left(x_{CT},z_{CT}\right)$ represents the coordinate associated with the lapping tool when *PEA* − 1 and PEA−2 receive $Z_{1}$ and $Z_{2}$ displacements, respectively. Additionally, $L_{x}$ denotes the half-length of the transfer beam. $x_{CT}$ , $z_{CT}$ and γ is represented by (1).


(1) $$\left\{ \begin{array}{l} x_{\mathrm{CT}}=z_{0}sin\gamma \\ \gamma =sin^{-1}(\frac{z_{2}-z_{1}}{2L_{x}})\\ z_{\mathrm{CT}}=L_{x}sin\gamma +z_{1}+z_{0}\cos\gamma \end{array}\right.$$

Substituting the second Equation in the first and third Equations:

(2) $$\left\{ \begin{array}{l} x_{CT}=z_{0}\frac{z_{2}-z_{1}}{2L_{x}}\\ z_{CT}=\frac{z_{1}+z_{2}}{2}+z_{0}\cos\left(sin^{-1}(\frac{z_{2}-z_{1}}{2L_{x}})\right) \end{array}\right.$$

The movement of the lapping tool is driven by two PZTs, which are excited by two sinusoidal signals with phase differences. The displacement of the piezoelectric actuators after being excited by the signal can be expressed as:

(3) $$\{\begin{array}{c} z_{1}=A\sin\left(2\pi ft\right)\\ z_{2}=B\sin\left(2\pi ft+\psi \right) \end{array}$$

where *f* is the frequency, *ψ* is the phase difference, *t* is the time, and *A* and *B* are the amplitudes of the two axes, respectively.

In order to easily express the trajectory of the lapping head, let A=B=U , Ψ=90° . Since the value of the input displacement is much smaller than the size and length of the transfer beam, substitute Equation (3) into (2),

(4) $$\{\begin{array}{c} x_{CT}=z_{0}\frac{U\sin\left(2\pi ft+\psi \right)-U\sin\left(2\pi ft\right)}{2L_{x}}\\ z_{CT}=\frac{U\sin\left(2\pi ft+\psi \right)+U\sin\left(2\pi ft\right)}{2}+z_{0}\cos\left(\sin^{-1}\left(\frac{U\sin\left(2\pi ft+\psi \right)-U\sin\left(2\pi ft\right)}{2L_{x}}\right)\right) \end{array}$$

According to the equivalent infinitesimal rule, Equation (4) can be expressed:

(5) $$\left\{ \begin{array}{l} \frac{x_{CT}}{z_{0}U/2L_{X}}=\sin(2\pi ft)-\cos(2\pi ft)\\ \frac{z_{CT}-z_{0}}{U/2}=\sin(2\pi ft)+\cos(2\pi ft) \end{array}\right.$$

The lapping head makes reciprocating motion in space, and its movement trajectory is elliptical. After calculation, the movement trajectory of the lapping head can be obtained as follows:

(6) $$\frac{x_{CT}^{2}}{{\left(z_{0}U/\sqrt{2}L_{x}\right)}^{2}}+\frac{(z_{CT}-z_{0}{)}^{2}}{{\left(U/\sqrt{2}\right)}^{2}}=1$$

According to the equation, it can be seen that the movement trajectory of the lapping head is an ellipse centered on $\left(0,z_{0}\right)$ .

### 3.2. The Simulation Analysis of VLD

VLD performance was analyzed using ABAQUS 2021 simulation software, and in order to avoid the damage of VLD during processing, the mode analysis and stress analysis were carried out. The material selected for the VLD was 7075Al, and the material parameters are shown in Table 1.

**Figure 4:**
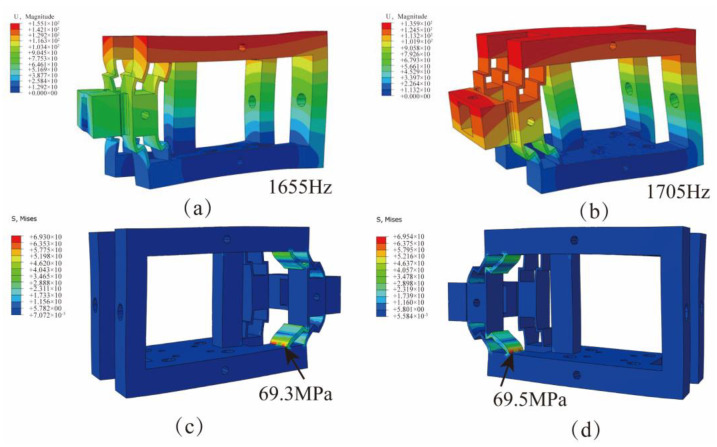
Performance test of vibration-assisted lapping device: (**a**) The first-order nature frequency (1655 Hz). (**b**) The second-order nature frequency (1705 Hz). (**c**) Stress distribution diagram on the right side of the device. (**d**) Stress diagram on the left side of the device.

During the simulation, the bottom surface of the device was completely fixed. The first two modes and the maximum stress are shown in Figure 4. The first two natural frequencies are 1655 Hz and 1705 Hz, respectively, as shown in Figure 4a,b. The operating frequency needs to be set smaller than the natural frequency. When a displacement load of 25 μm was applied on both sides of the device, the maximum stresses on both sides of the device were 69.3 MPa and 69.5 MPa, respectively, lower than the allowable stress of the material, as shown in Figure 4c,d. Therefore, the device could meet the normal working requirements.

## 4. Simulation of NVL for SiCp/Al Composites

The material structure of SiCp/Al has heterogeneous characteristics, and the difference in elastic modulus and hardness between aluminum matrix and SiC particles leads to surface defects during machining. Therefore, an NVL SiCp/Al composite material model is established in this section. The removal mechanism of SiCp/Al composite during diamond particle lapping, the form of surface defects, and the effect of parameters (vibration amplitude *A*, vibration frequency *f*, lapping speed *v_c_*, lapping depth *a_p_*) on the surface morphology were analyzed by finite element simulation method.

### 4.1. Finite Element Model Setting of NVL for SiCp/Al Composites

**Figure 5:**
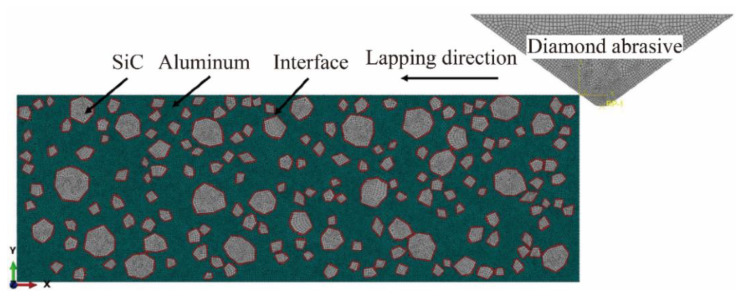
The assembly drawing of abrasive and workpiece.

The formation process of surface defects in the NVL process and the influence of process parameters on surface defects were analyzed by simulation. A SiCp/Al composite material model with SiCp/Al material, diamond abrasive particles, and a two-phase interface was established, as shown in Figure 5. The two-phase interface was used to reflect the stick-slip effect between SiC particles and the Al matrix. A cohesive element with a thickness of 0 was embedded between SiC particles and the Al matrix to simulate the two-phase interface. The volume fraction and averaged particle size of SiC are 20% and 10 μm, respectively. The abrasive material was diamond, and the effect of lapping heat and abrasive wear on the processing was not considered. The diamond abrasive was simplified into a triangle with a peak angle of 120° and set as a rigid body. A velocity load was applied to the abrasive, and the bottom and left side of the workpiece are fixed constraints.

The plastic deformation phenomenon occurring during processing can be represented by the Johnson–Cook constitutive model [31]. The material properties of Aluminum and SiC are shown in Table 2.

### 4.2. Simulation Study on SiCp/Al Removal Characteristics of NVL

**Figure 6:**
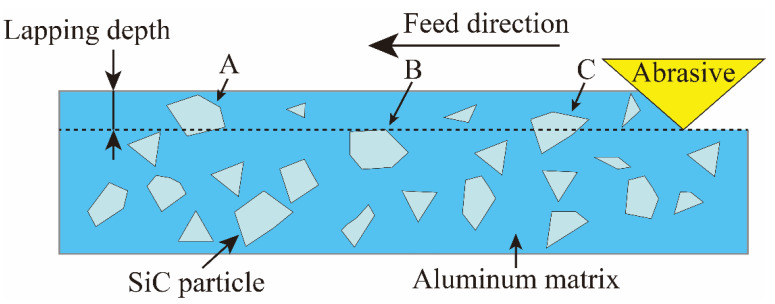
Three positions of abrasive particles and SiC particles.

Due to the special structure of SiCp/Al, the shear position of SiC particles will directly affect the generation of surface defects. As shown in Figure 6, the shear position of the SiC particle can be divided into three cases: the abrasive is located above the SiC particle (Position A), the abrasive is located in the middle of the SiC particle (Position C), and the abrasive is located below the SiC particles (Position B).

**Figure 7:**
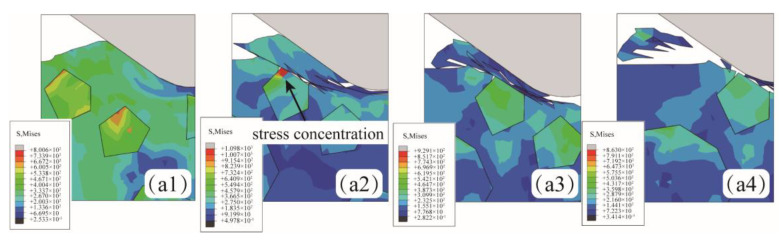
The abrasive is located above the SiC particle (**a1**–**a4**).

As shown in Figure 7(a1–a4), when the abrasive was located above the SiC particle (Position A), slight stress concentration occurred in the SiC particle, but no particle breakage occurred. As the abrasive continued to move, the aluminum matrix became stress concentration and plastic deformation due to the impact and extrusion of the abrasive. The increasing stress leads to the formation of cracks and eventually extends to the surface of the workpiece. Although the stress concentration of SiC particles occurred, particle shedding and interfacial debonding did not occur, and the chip is mainly composed of an aluminum matrix.

**Figure 8:**
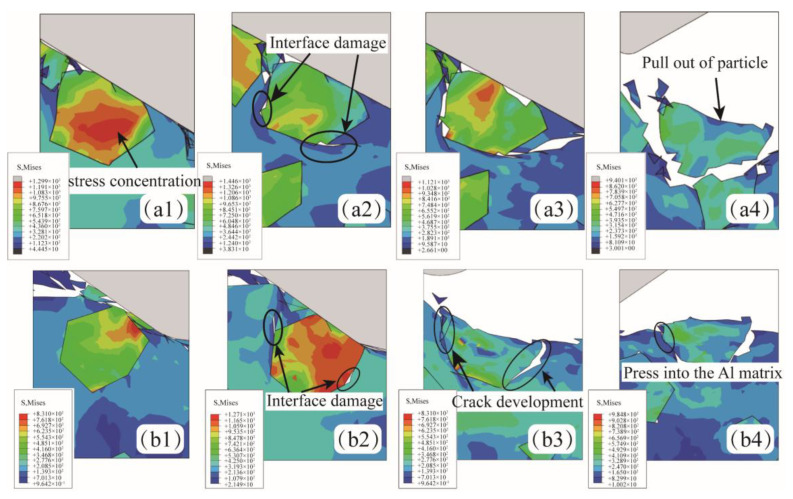
The abrasive is located in the middle of the SiC particle. (**a1**–**a4**) The SiC particles shed from the aluminum matrix. (**b1**–**b4**) The SiC particles were pressed into the aluminum matrix.

Figure 8(a1–b4) shows the formation of particle breakage and particle shedding when SiC particles are located at position C. Figure 8(a1–a4) shows the phenomenon of particle shedding under shear action. After abrasive contact with SiC particles, stress concentration occurs in the central region of SiC particles. The large stress is released through aluminum matrix tearing and interfacial debonding. Under the action of extrusion, the interface completely fails, and SiC particles fall off. Figure 8(b1–b4) shows the particle tumbling process of SiC particles. Under the action of cohesive force and extrusion pressure, the interface damage occurs due to stress concentration, and the interface damage expands continuously under the action of lapping force. However, the SiC particles do not entirely fall off but are deflected at an angle and pressed into the aluminum matrix, as shown in Figure 8(b4). It is worth noting that in this state, the SiC and aluminum matrix bonding area is small, the state is unstable, and it is prone to subsequent processing and fall-off.

**Figure 9:**
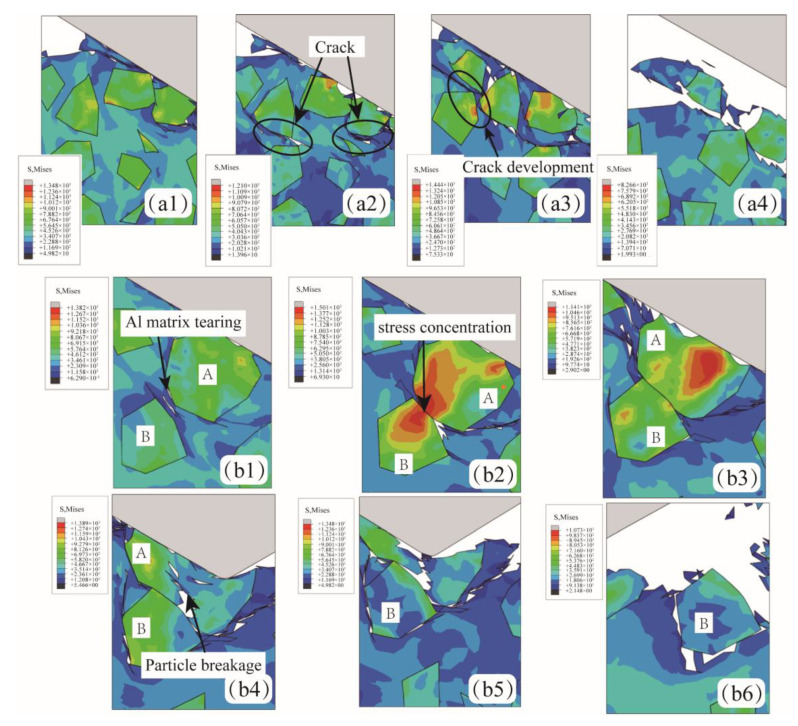
The abrasive is located below the SiC particle. (**a1**–**a4**) The SiC particles were removed together with the aluminum matrix.(**b1**–**b6**) The SiC particles were crushed. (A and B represent SiC particles).

Figure 9(a1–a4) shows the formation process of surface defects when SiC particles are located at position A. The lapping depth is greater than the SiC particle size in this case. The cracks preferentially form in the aluminum matrix with a lower yield limit, expanding and connecting under continuous stress. Finally, the lapping chips composed of the aluminum matrix and SiC particles are formed. Figure 9(b1–b6) shows the process of SiC particles breaking under the effect of lapping force, which is more likely to occur when two SiC particles are close together. As shown in Figure 9(b1), the aluminum matrix deforms due to the low hardness and the pressing of SiC particles. The proximity of adjacent SiC particles to each other causes the surrounding aluminum matrix to be torn. The particles are in direct contact and produce a sizeable, concentrated stress at the contact position, as shown in Figure 9(b2). When the internal stress exceeds the yield limit of SiC particles, particle A produces a crack, as shown in Figure 9(b3,b4). As the extrusion degree intensifies, the crack in particle A propagates through particle A, and the particle is broken, as shown in Figure 9(b5,b6).

### 4.3. Influence of Machining Parameters on SiCp/Al Lapping Surface Quality

**Figure 10:**
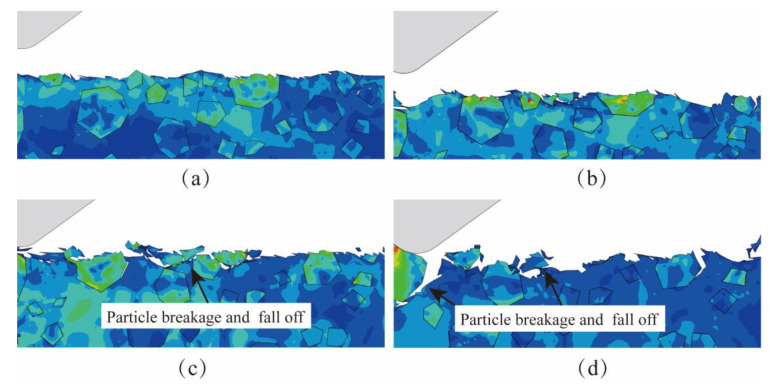
The simulation results of different lapping depths. (**a**) The lapping depth *a_p_* is 4 μm. (**b**) The lapping depth *a_p_* is 6 μm. (**c**) The lapping depth *a_p_* is 8 μm. (**d**) The lapping depth *a_p_* is 10 μm.

Figure 10 shows the simulation comparison of the surface morphology of NVL SiCp/Al at different lapping depths *a_p_*. When *a_p_* is 4 μm, the lapping depth is small, and the abrasive removes lesser material. The lapping force is small, and the removal state remains stable, leading to better surface uniformity and surface quality after processing, as depicted in Figure 10a. As the *a*_p_ increases to 6 μm or 8 μm, the removal depth of the abrasive increases, and the phenomena of interfacial debonding and particle breakage increase. Moreover, some particles are more likely to fall off and experience interfacial debonding because of their weak bonding degree with the aluminum matrix. The SiC particles form pits on the surface after falling off. This phenomenon is particularly noticeable when the lapping depth increases to 8 μm, as shown in Figure 10b,c. When the *a*_p_ increases to 10 μm, particle fracture and shedding increase significantly, as shown in Figure 10d. In summary, excessive lapping depth causes SiC particle fracture, and the SiC particles fall out more obviously.

**Figure 11:**
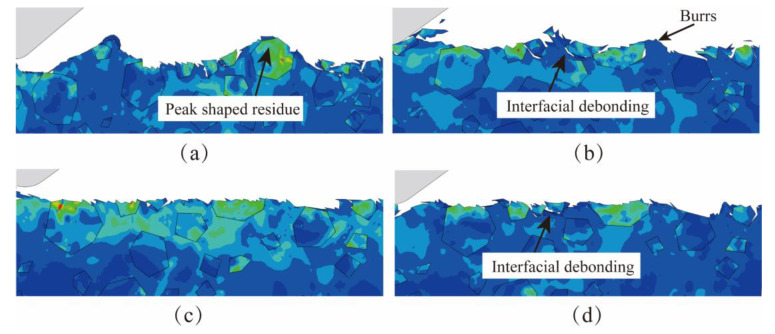
The simulation results of different lapping speeds. (**a**) The lapping depth *v_c_* is 12 mm/min. (**b**) The lapping depth *v_c_* is 19 mm/min. (**c**) The lapping depth *v_c_* is 25 mm/min. (**d**) The lapping depth *v_c_* is 31 mm/min.

Figure 11 shows the simulation comparison of NVL SiCp/Al surface morphology when the lapping speed *v_c_* changes. Because the two-dimensional single-abrasive lapping simulation cannot show all the velocity characteristics of abrasive, the lapping speed *v_c_* in the simulation mainly represents the synthesis velocity of abrasive. When *v_c_* is 12 mm/min, it can be observed that there are peaks, as shown in Figure 11a. When *v_c_* increases to 19 mm/min, the peak-shaped residue on the machined surface disappears, and the machined surface becomes flat, but there are still burrs on the surface, as shown in Figure 11b. When *v_c_* is 25 mm/min, there are no obvious defects on the surface of the workpiece, and the phenomena of particle shedding and interfacial debonding are reduced, as shown in Figure 11c. When *v_c_* increases to 31 mm/min, the phenomena of particle breakage, interfacial debonding, and particle shedding occur on the workpiece surface. In conclusion, With the increased lapping speed, the surface defect becomes better first and then worse. The surface defects are the least at *v_c_* = 25 mm/min, and the particle fracture and the interfacial debonding are lesser.

**Figure 12:**
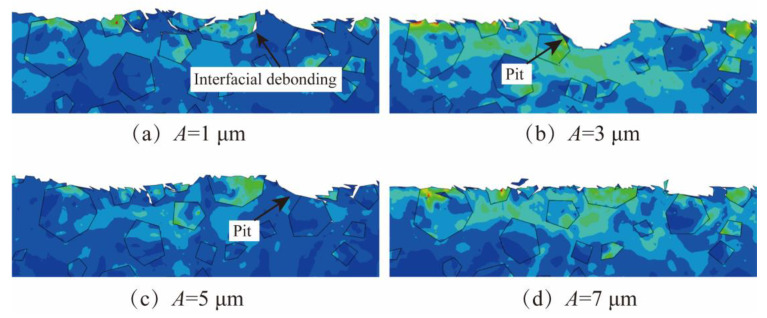
The simulation results of amplitude. (**a**) The lapping depth *A* is 1 μm. (**b**) The lapping depth *A* is 3 μm. (**c**) The lapping depth *A* is 5 μm. (**d**) The lapping depth *A* is 7 μm.

Figure 12 shows the simulation comparison of NVL SiCp/Al surface morphology when the amplitude A changes. As seen in Figure 12a, when *A* is 1 μm, the surface after lapping is uneven, with more burrs, some fractured SiC particles, and obvious interface disbanding between particles and matrix. When amplitude *A* = 3 μm, large pits are formed after significant damage to SiC particles in a large area, as shown in Figure 12b. At an amplitude of *A* = 5 μm, although there are still burrs on the surface, the pits are significantly smaller compared to amplitudes *A* = 1 μm or *A* = 3 μm, as shown in Figure 12c. With the amplitude *A* = 7 μm, interfacial debonding and pits between particles and matrix reduces, as shown in Figure 12d.

In conclusion, surface defects are more apparent when the amplitude is less than 7 μm. This is because the separation characteristics of lapping processing are not prominent, and the vibration processing characteristics could not be fully utilized. Additionally, the chips generated during the processing are difficult to discharge, resulting in the accumulation of chips in the abrasive front end, which affects the processing effect. The larger amplitude highlights the advantages of vibration processing. With the increase in amplitude, the motion trajectory of the abrasive changes, and the periodic contact process between the abrasive and the workpiece becomes significant, which is conducive to the discharge of lapping chips, and the surface quality after processing is also significantly improved.

**Figure 13:**
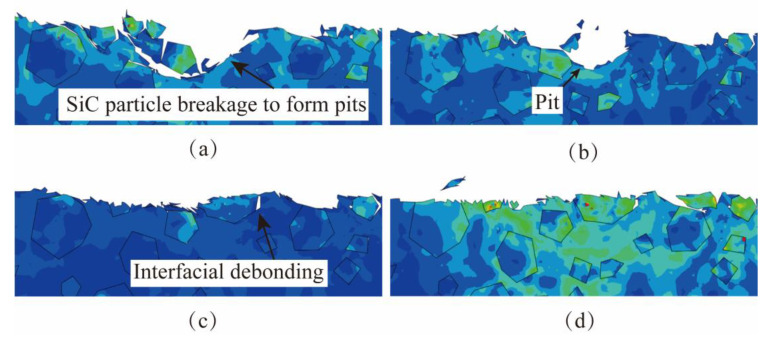
The simulation results of frequency. (**a**) The lapping depth *f* is 0 Hz. (**b**) The lapping depth *f* is 150 Hz. (**c**) The lapping depth *f* is 300 Hz. (**d**) The lapping depth *f* is 450 Hz.

Figure 13 shows the simulation comparison of lapping under different vibration frequencies, where the frequency *f* is 0 Hz, 150 Hz, 300 Hz, and 450 Hz, respectively. When *f* is set to 0 Hz (conventional lapping method) or 150 Hz, there are apparent large pits on the surface of the workpiece, which are formed after the SiC particles fall off, as shown in Figure 13a,d. When the frequency *f* is increased to 300 Hz, some small pits and burrs still exist on the surface. However, particle fracture and detachment occurrences are significantly reduced, improving surface topography, as shown in Figure 13c. Increasing the frequency *f* to 450 Hz eliminates noticeable pits on the workpiece surface, reduces shedding between the particle and matrix interface, and results in a flat and uniform surface with improved surface quality, as shown in Figure 13d. In conclusion, applying vibration in conjunction with CL processing proves beneficial in enhancing surface quality. The effect of low-frequency vibration on surface defects is weak. Increasing the frequency contributes to further improvements in surface quality and reduced occurrences of particle fracture and debonding.

## 5. Test of Vibration-Assisted Lapping Device and Lapping Experiment

In this section, firstly, the VLD test system is built, and its resolution, stroke, natural frequency, and motion trajectory are tested. Then, based on the designed VLD, an NVL SiCp/Al composite experimental system was built, and explored the effects of vibration frequency *f*, amplitude *A*, spindle speed *n*, feed speed *v_f_*, and lapping depth *a_p_* on the lapping force and workpiece surface roughness by SiCp/Al composite NVL processing experiment.

### 5.1. Performance Test of VLD

**Figure 14:**
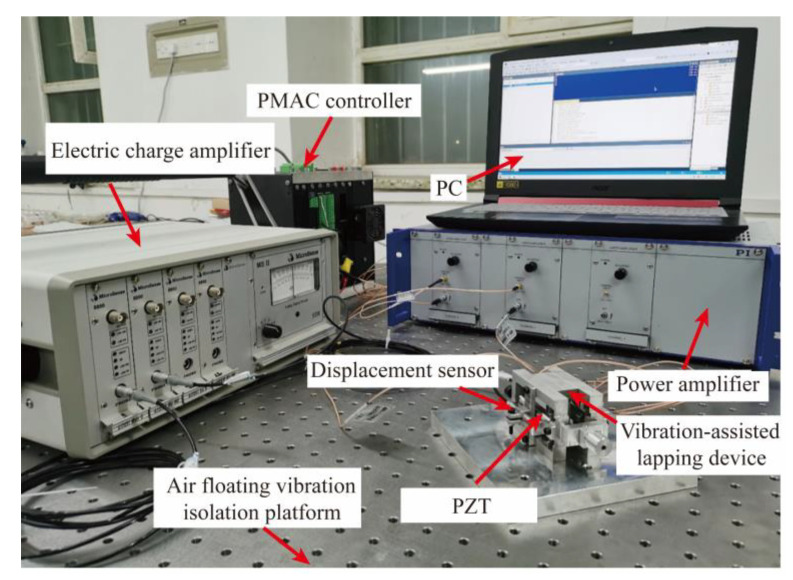
The test system of VLD.

The VLD test system was built, and the VLD test experiment was carried out, which mainly included the natural frequency test, resolution test, output displacement test, and motion track acquisition. Figure 14 shows the performance test system of the VLD. The experimental instruments used in the test mainly include a piezoelectric actuator (PZT, COREMORROW Inc. Harbin, China), controller (Power PMAC), power amplifier (E-500, PI Inc. Shanghai, China), displacement sensor (2805, MicroSense Inc., Lowell, MA, USA), charge amplifier (DE 5300-013, MicroSense Inc.). The power amplifier amplifies the signal sent by the controller and acts as the drive signal of the piezoelectric actuator. The piezoelectric actuator outputs the displacement after receiving the signal; the displacement sensor collects the output displacement signal, transmits it to the controller, and outputs the displacement at the end.

**Figure 15:**
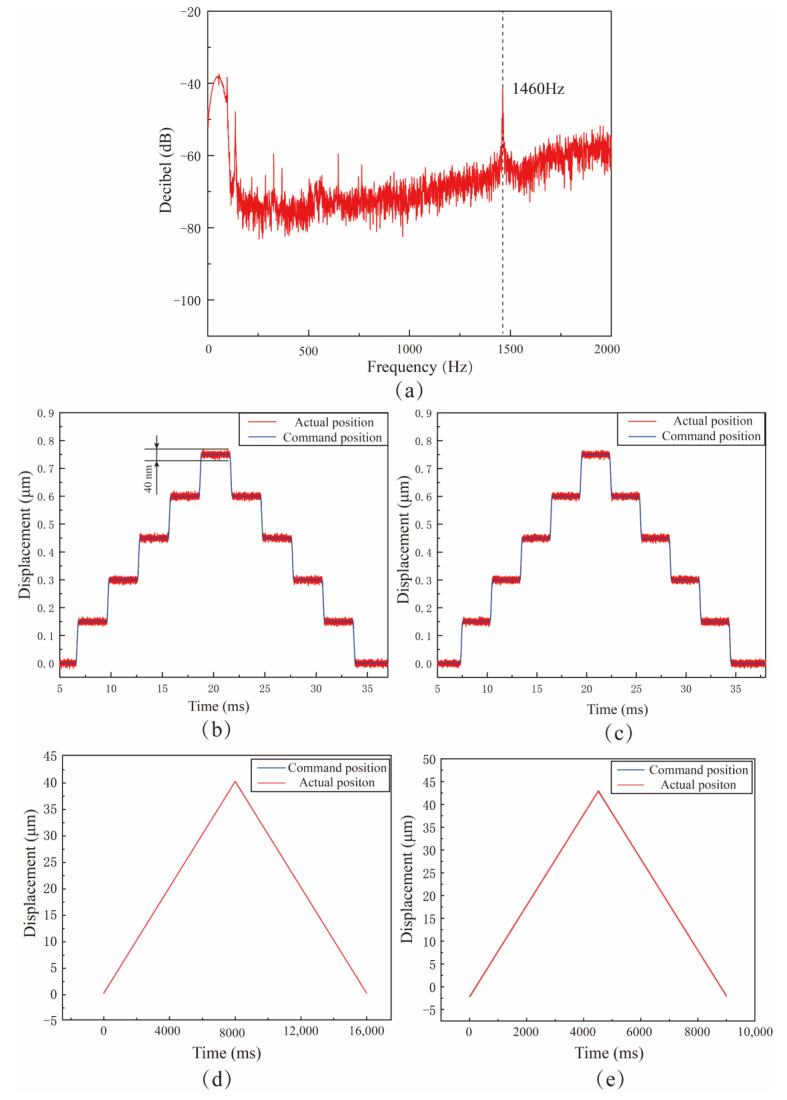
The test results of VLD: (**a**) natural frequency test results. (**b**) Z_1_ axis resolution test results. (**c**) Z_2_ axis resolution test results. (**d**) Z_1_ axis displacement test results. (**e**) Z_2_ axis displacement test results.

The natural frequency, movement range, and resolution are the parameters that must be paid attention to. In order to avoid damage to the device, the VLD needs to operate at a frequency less than the first-order natural frequency. As shown in Figure 15a, the natural frequency of the VLD is 1460 Hz, which is slightly lower than the finite element simulation result of 1655 Hz. Due to machining errors and different contact conditions, there are usually some differences between the performance of the actual machining device and the simulation results under the ideal situation. The parameter range of VLD under working state should be determined by referring to the results of actual performance tests. Motion resolution significantly affects the control effect of the VLD. As shown in Figure 15b,c, a step signal is applied to two axes of the VLD, and the output displacement is measured by a charge sensor. As shown in Figure 15d,e, test results show that the resolution of both axes of the device can reach 40 nm. A triangular wave is used to test the output stroke of the VLD. Test the output displacement of the vibration-assisted lapping device with triangular waves and continuously increase the amplitude of triangular waves. Finally, the limit travel of the z1 axis and z2 axis of the vibration-assisted lapping device is 40 nm and 45 nm, respectively, which can realize fast-tracking of the input signal and meet the amplitude requirements in the machining process.

**Figure 16:**
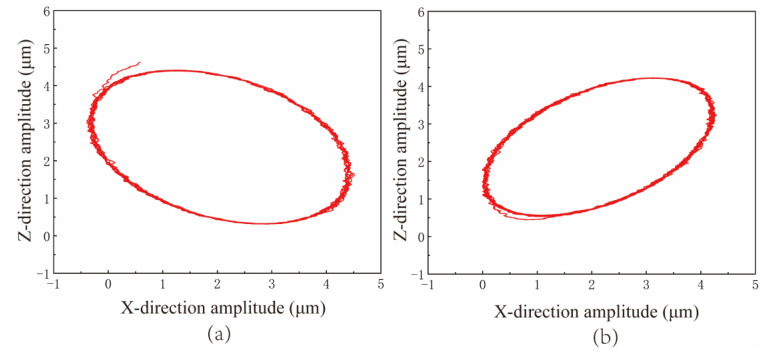
The movement trajectory of VLD under different phase differences: (**a**) 45°phase difference. (**b**) 90°phase difference.

In the NVL process, the output of the device vibration track has a crucial impact on the lapping process. In order to determine the shape and stability of the device output track, the device trajectory output test is carried out. Sinusoidal signals with 45° and 90° phase differences are applied to the piezoelectric brake. Figure 16a,b shows the output trajectories of the device when the input signals are 45° and 90° phase differences, respectively. The output trajectories of the device under the phase differences of 45° and 90° are elliptical, and the trajectory outline is clear. It can meet the demand of motion trajectory output during processing.

### 5.2. Experiment on NVL of SiCp/Al Composites

**Figure 17:**
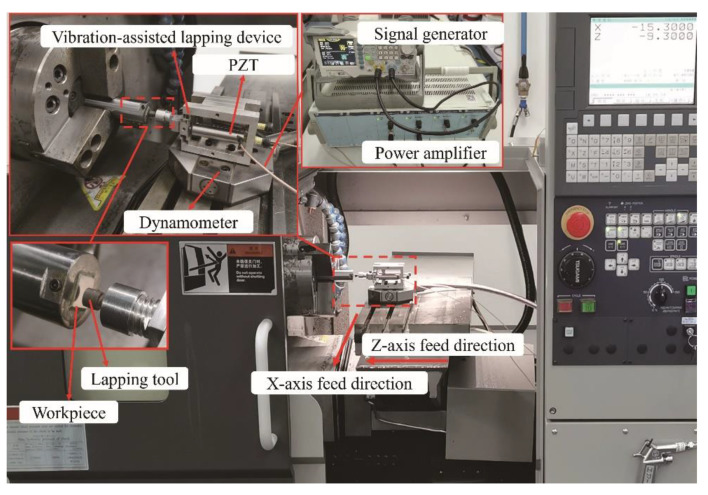
SiCp/Al composite material NVL experimental system.

Figure 17 shows the NVL experimental system of SiCp/Al. During the experiment, the dynamometer (9109, Kistler Inc. Winterthur, Switzerland) is installed on the machine guide rail platform, the VLD is installed on the dynamometer, the grinding head is installed in the front end of the VLD, and the machine guide rail does X-direction feed movement. A signal generator and power amplifier control the vibration parameters. SiCp/Al is 10 × 10 × 10 mm, and the average size and volume fraction of SiC are 10 μm and 20%, respectively. Each workpiece is pretreated before the experiment. Each experimental parameter is repeated three times, and the experimental parameters are shown in Table 3 and Table 4. The surface roughness of five random points on the polished surface was measured using a white-light interferometer (NewView 8000, Zygo Inc. Middlefield, CT, America).

### 5.3. Effect of Process Parameters

#### 5.3.1. Comparison of NVL and CL

**Figure 18:**
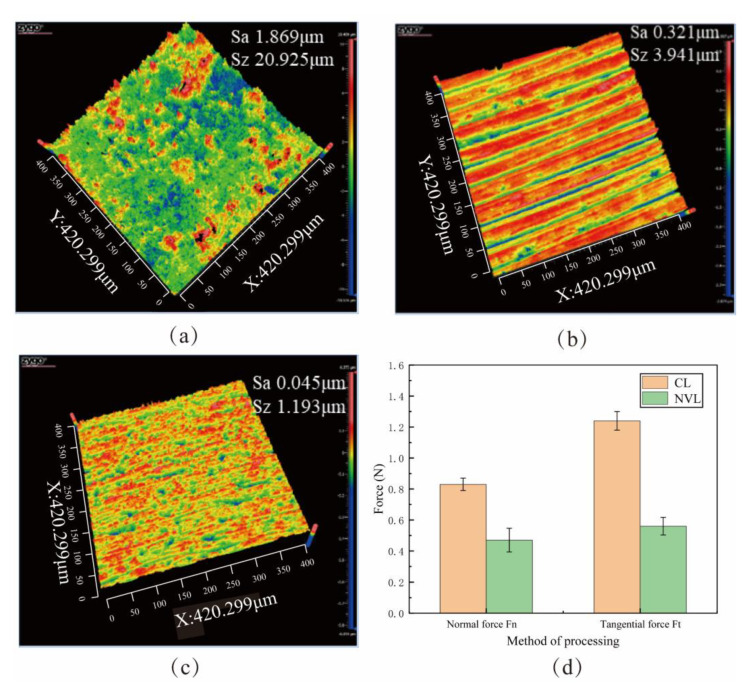
The surface roughness of the workpiece: (**a**) Original surface roughness. (**b**) Conventional lapping surface roughness. (**c**) Non-resonant vibration-assisted lapping surface roughness. (**d**) Lapping force of NVL and CL.

In order to study the difference between surface quality and lapping force after NVL and CL, the NVL experiment and CL experiment are conducted, respectively, and experimental parameters are shown in Experiment 1 and Experiment 2 in Table 3. Figure 18 shows the lapping force and the surface topography of the workpiece. As shown in Figure 18a,b, the abrasive of the CL method has a single motion path, and the removal process is continuous. There is a significant plow effect on surfaces using CL methods, and lapping debris in the lapping area is difficult to discharge, resulting in increased lapping force. The surface quality decreases from 1.869 μm to 0.321 μm. The NVL method has less lapping force, and the surface roughness is reduced from 1.869 μm to 0.045 μm, which is better than the CL method, as shown in Figure 18c,d.

**Figure 19:**
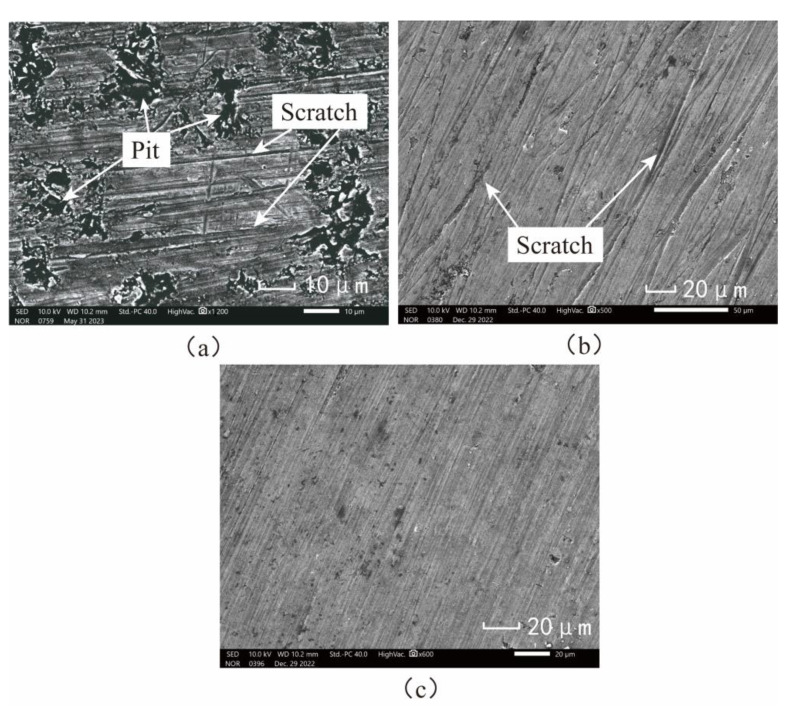
Surface Topography of the workpiece. (**a**) Original surface topography of the workpiece. (**b**) Conventional lapping surface morphology. (**c**) Non-resonant vibration-assisted lapping surface morphology.

Figure 19a shows the initial surface topography. The surface defects are mainly pits and scratches. Due to the difference in brittleness and plasticity between SiC particles and aluminum matrix, the SiC particles are more prone to brittle crushing and forming pits during processing. The microscopic surface of SiCp/Al processed by CL method is shown in Figure 19b. There are apparent scratches on the surface, but the pits disappear, which is because the aluminum matrix is coated on the surface after melting. The lapping chips are difficult to discharge, and lapping force and lapping heat increase. All these effects hurt the surface quality of the workpiece [32,33,34]. The microscopic morphology of SiCp/Al processed by the NVL method is shown in Figure 19c. The scratches on SiCp/Al surface are significantly reduced. The above phenomena indicate that the application of vibration reduces surface damage.

#### 5.3.2. Effect of Spindle Speed n

**Figure 20:**
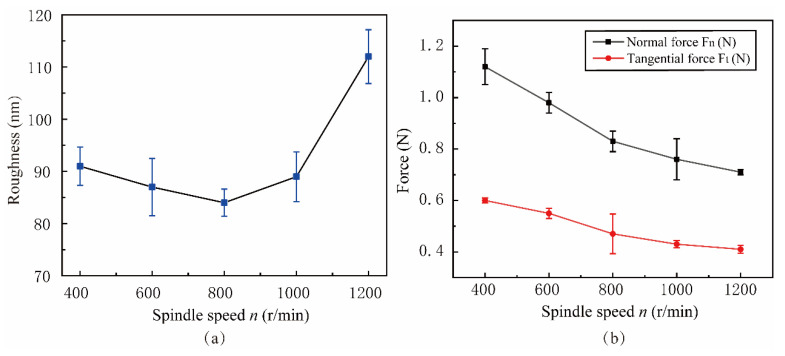
Effect of spindle speed on surface roughness and lapping force: (**a**) Roughness, (**b**) Force.

Figure 20a shows the influence of surface roughness with *n*. Figure 20b shows the law of lapping force variation in the spindle speed *n* range of 400 r/min to 1200 r/min. The experimental parameters are shown in Experiment 3 of Table 3. The surface roughness S_a_ shows a trend of first decreasing and then increasing, which is similar to the trend of the simulation analysis results, and the surface roughness S_a_ = 0.085 μm is the lowest when *n* = 800 r/min. The normal and tangential lapping force of NVL shows a decreasing trend. This phenomenon is because the change in spindle speed affects the tool wear, lapping force, and lapping trajectory. Because the spindle speed *n* is inversely proportional to the lapping depth and proportional to the lapping path length per unit time, the lapping force and friction resistance decrease with the increase of the spindle speed, and the surface quality is improved. When *n* exceeds 800 r/min, the wear of lapping tools is intensified due to excessive speed, and the temperature in the lapping area increases, resulting in the lapping chips becoming soft and sticking to the machined area. The secondary scratch of lapping chips increases the roughness of the machined surface.

#### 5.3.3. Effect of Feed Speed v_f_

**Figure 21:**
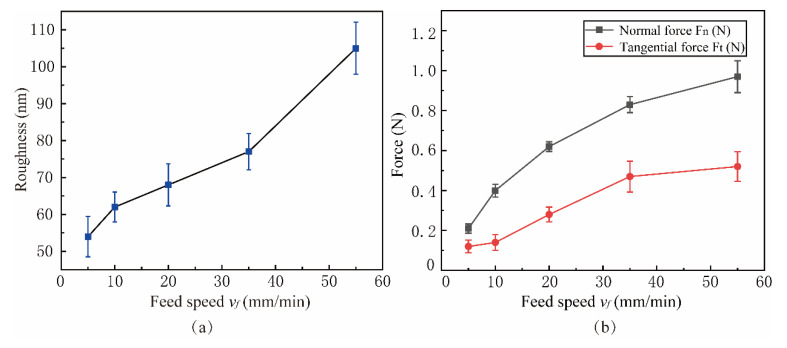
Effect of feed speed on surface roughness and lapping force: (**a**) Roughness, (**b**) Force.

Figure 21 shows the influence of feed speed *v_f_* on surface roughness and lapping force in the range of 5 mm/min to 55 mm/min. The experimental parameters are shown in Experiment 4 of Table 3. It can be seen from Figure 21a that the surface roughness is proportional to the feed speed, and the point corresponding to the best surface roughness is *v_f_* = 5 mm/min because the high feed rate causes the workpiece surface to be unable to be fully lapping. At the same time, the material removal rate and lapping force of a single abrasive increases, resulting in more surface damage to SiCp/Al and an increase in SiC particle shedding and particle fracture on the machined surface, which affects the surface quality after machining. The difference between experimental and simulation results of feed speed *v_f_* may be due to different parameter Settings, and the abrasive motion path of the NVL method is dependent on the spindle speed [24,35].

#### 5.3.4. Effect of Lapping Depth a_p_

**Figure 22:**
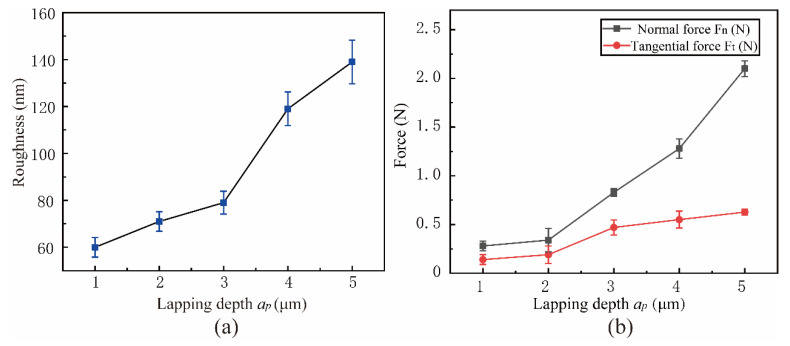
Effect of lapping depth on surface roughness and lapping force: (**a**) Roughness, (**b**) Force.

Figure 22 shows the influence of surface roughness and lapping force in the range of lapping depth *a_p_* from 1 μm to 5 μm. The experimental parameters are shown in Experiment 5 of Table 3. With the increase of *a_p_*, surface roughness and lapping force increase, which is consistent with the simulation results. When the lapping depth *a_p_* = 1 μm, the surface roughness reaches the minimum value of 60 nm, and the lapping force is also the minimum value. The reason for this phenomenon is that when the *a_p_* is small, the SiC particle fracture and particle shedding phenomenon in the processing process is lesser, the lapping chips are also small, and the fine debris is more easily discharged from the processing area under the action of vibration. The contact area between the abrasive and workpiece will increase with the increased lapping depth, resulting in increased chip deformation force and friction, and SiC particles are prone to crushing and particle pulling out phenomenon. As the lapping depth is close to the amplitude of the lapping tool, the advantages of NVL are weakened, and the discharge of grind chips is difficult and accumulates between the workpiece and the lapping head, causing lapping force and heat increase, resulting in a reduction in surface quality.

#### 5.3.5. Effect of Vibration Frequency f

**Figure 23:**
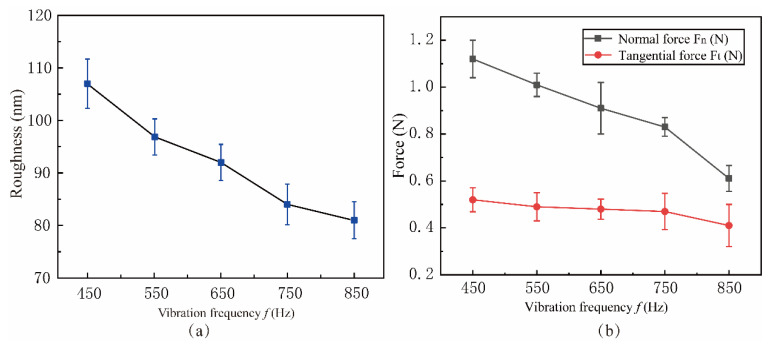
Effect of vibration frequency on surface roughness and lapping force: (**a**) Roughness, (**b**) Force.

Figure 23 shows the variation of surface roughness and lapping force with vibrational frequency *f* between 450 Hz and 850 Hz. The experimental parameters are shown in Experiment 6 of Table 3. When the vibration frequency *f* = 850 Hz, the surface roughness reaches the minimum value of 81 nm, and the vibration frequency is inversely proportional to the lapping force. According to the simulation results of the frequency factor, the periodic separation process of abrasive and workpiece is not apparent at low-frequency vibration, and the suppression effect on surface defects is poor. High-frequency vibration enhances the effect of abrasive periodically impacting the workpiece, and the material removal volume within a single vibration cycle decreases. The number of chips discharged with vibration per unit time also increases, reducing the attachment and bonding of lapping chips, thereby delaying tool wear and reducing lapping force and surface roughness.

#### 5.3.6. Effect of Vibration Amplitude A

**Figure 24:**
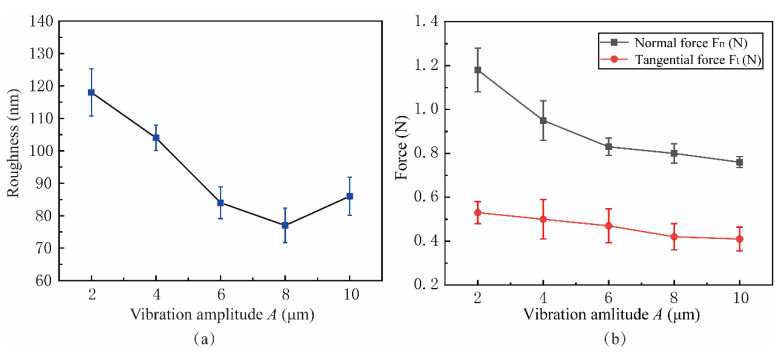
Effect of vibration amplitude on surface roughness and lapping force: (**a**) Roughness, (**b**) Force.

Figure 24 shows the influence of lapping force and surface roughness in the vibration amplitude *A* range from 2 μm to 10 μm. The experimental parameters were shown in experiment 7 of Table 3. When *A* = 8 μm, the lowest surface roughness value is 0.077 μm. Before the amplitude *A* reaches 8 μm, the surface quality is inversely proportional to the amplitude. This is because the vibration amplitude affects the contact separation process of the abrasive. The low amplitude is not conducive to the removal of lapping chips and the reduction of surface defects, which is consistent with the trend of simulation results. After the *A* exceeds 8 μm, the impact of the abrasive on the workpiece is too strong, increasing surface roughness. The lapping force decreases with the increase of *A*, as shown in Figure 24b, which is because the increase in amplitude makes the contact–separation effect more apparent, and the lapping chips can be discharged more smoothly, thus reducing the lapping force during the lapping process.

### 5.4. Analysis of Lapping Surface Micro-Morphology

From the simulation analysis in Section 4, there will be interfacial debonding, particle fracture, particle tumbling, and particle fracture of SiC particles in the lapping process, which will cause damage to the workpiece surface. The surface of the SiCp/Al workpiece after lapping is detected and analyzed by an electron microscope, and the results are compared with the phenomena in the simulation to verify the accuracy of the simulation.

**Figure 25:**
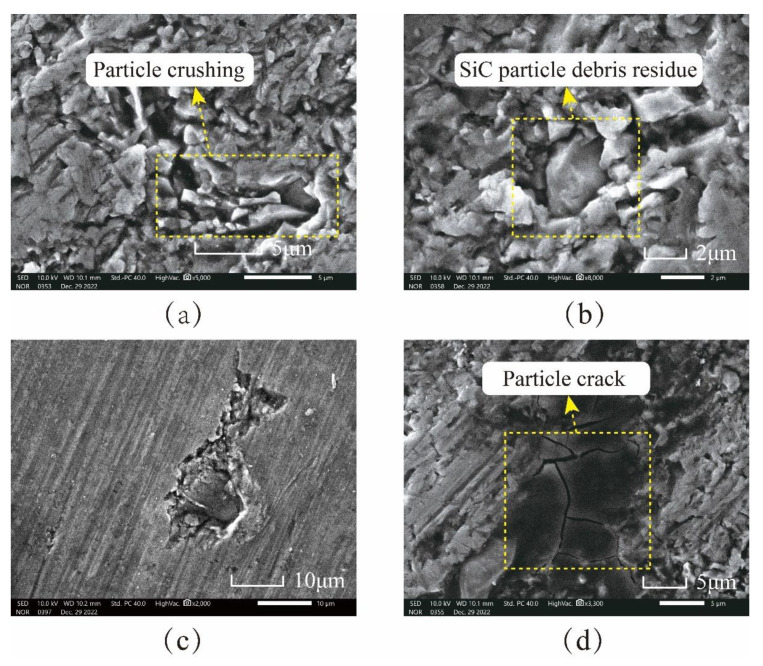
Morphology of SiC particles on workpiece surface after lapping: (**a**) SiC particle fracture; (**b**) the SiC particulate debris remained in the matrix; (**c**) SiC particle tumbling; (**d**) the SiC particles produce cracks.

Figure 25 shows the microscopic surface topography of SiC particles after lapping by electron microscope. After complete fracture, SiC particles are not separated from the matrix. They are still embedded in the matrix to form SiC particle clusters in Figure 25a because SiC particles generate huge internal stress concentrations under the action of the lapping tool. When the vibrating lapping head passes through SiC particles with severe stress concentration, SiC particles are completely fractured under impact and extrusion, and the fracture particles are squeezed again and then converge to form clusters. As shown in Figure 25b, SiC particles are crushed, and some particles remain in the aluminum matrix. As shown in Figure 25c, the SiC particles did not immediately break away from the surface after shedding with the matrix. However, they tumbled on the surface of the workpiece through the extrusion and pushing of the lapping head and are embedded in the aluminum matrix after leaving a period of plow marks on the surface of the workpiece. The remaining lapping chips will likely fall off in this state and eventually form pits. As shown in Figure 25d, under the action of lapping force, cracks occur in the SiC particles and further expand and finally penetrate the SiC particles.

## 6. Conclusions

A VLD is designed and tested. The NVL SiCp/Al process is studied using the VLD. The removal characteristics of the NVL SiCp/Al process are analyzed. Single-factor experiments were carried out on the process parameters such as spindle speed *n*, feed speed *v_f_*, lapping depth *a_p_*, vibration frequency *f*, and vibration amplitude *A*. The following conclusions can be drawn:

1. A VLD is designed. The motion trajectory of the VLD is analyzed, and its modal and stress distribution are simulated by the simulation method. The test results show that the first-order natural frequency is 1460 Hz, the resolution is 40 nm, and the two-axis stroke is 45 μm and 40 μm, respectively;
2. NVL SiCp/Al can reduce the lapping force. Spindle speed, vibration amplitude, and vibration frequency are inversely correlated with lapping force, and the lapping force reaches the minimum value at *f* = 900 Hz, *A* = 10 μm and *n* = 1200 r/min, respectively. The feed speed and lapping depth positively correlate to the lapping force;
3. NVL lapping SiCp/Al effectively reduces the surface roughness. The vibration amplitude and spindle speed decreased first, then increased, reaching the minimum values at *A* = 8 μm and *n* = 800 r/min, respectively. The lapping depth and feed speed were inversely correlated with the surface roughness. Reaches the minimum values of 0.054 μm and 0.059 μm at *v_c_* = 5 mm/min and *a_p_* = 1 μm, respectively. The vibration frequency is proportional to the surface roughness and reaches a minimum of 0.081 μm at *f* = 900 Hz. The changing trend of process parameters in machining experiments is similar to that in simulation.

## Figures and Tables

**Table 1:** The mechanical properties of the 7075Al.

Elastic Modulus E (GPa)	Density ρ (kg·m^−3^)	Poisson’s Ratio μ	Yield Strength σ (MPa)
71.7	2810	0.33	503

**Table 2:** The physical and mechanical properties of 6005 aluminum and SiC particles.

Materials	Sic Particles	6005 Al
Young’s modulus (GPa)	183	70
Poisson’s ratio	0.2	0.3
Density (kg/m^3^)	3163	2700
Thermal conductivity (W/mk)	81	190
Coefficient of thermal expansion (K^−1^)	4.9 × 10^−6^	2.3 × 10^−5^
specific heat capacity (J/gk)	0.427	0.91

**Table 3:** Experimental variable parameter setting of NVL.

Experimental Variable					
Experiment Number	Spindle Speed *n* (r/min)	Feed Speed *v_f_* (mm/min)	Lapping Depth *a_p_* (μm)	Vibration Frequency *f* (Hz)	Vibration Amplitude *A* (μm)
Experiment 1	800	5	1	0	0
Experiment 2	800	5	1	850	8
Experiment 3	400, 600, 800, 1000, 1200	35	3	750	6
Experiment 4	800	5, 10, 20, 35, 55	3	750	6
Experiment 5	800	35	1, 2, 3, 4, 5	750	6
Experiment 6	800	35	3	450, 550, 650, 750, 850	6
Experiment 7	800	35	3	750	2, 4, 6, 8, 10

**Table 4:** Experimental controlled variable parameter setting of NVL.

Controlled Variable	
Diamond abrasive size (μm)	5
Lapping time t (s)	30
Initial surface roughness S_a_ (μm)	1.869
Diameter of lapping head (mm)	6

## Data Availability

The data that support the findings of this study are available from the corresponding author upon reasonable request.
